# Supplementary material for: Massively parallel cantilever-free atomic force microscopy
Source: Nat Commun. 2021 Jan 15;12:393. doi: 10.1038/s41467-020-20612-3 (PMC7810748; doi:10.1038/s41467-020-20612-3)
Supplement: Supplementary file 1 — Supplementary Information [file 41467_2020_20612_MOESM1_ESM.pdf]

Supplementary Information

for

Massively Parallel Cantilever-free Atomic Force Microscopy

Wenhan Cao[1], Nourin Alsharif[1], Zhongjie Huang[2], Alice E. White[1,3,4,5], YuHuang Wang[2] and Keith A. Brown[1,3,5,*]

[1]Department of Mechanical Engineering, Boston University, Boston, MA, 02215, USA
[2]Department of Chemistry and Biochemistry, University of Maryland, College Park, MD, 20742, USA
[3]Division of Materials Science & Engineering, Boston University, Boston, MA, 02215, USA
[4]Department of Biomedical Engineering, Boston University, Boston, MA, 02215, USA
[5]Physics Department, Boston University, Boston, MA, 02215, USA
[*]Correspondence to be addressed to brownka@bu.edu

Supplementary Note 1. Model of the Distributed Optical Lever **2**
Supplementary Note 2. Estimation of Probe and Sample Deformation **5**
Supplementary Note 3. Mechanical Characterization of Cantilever-free Probes **8**
Supplementary Note 4. Estimation of Crosstalk **10**
Supplementary Note 5. Optomechanics of Cantilever-free Probes **11**
Supplementary Note 6. Quantification of Repeatability **12**
Supplementary Note 7. Observing Lateral Forces with Cantilever-free Probes **13**
Supplementary References **14**

Supplementary Note 1. Model of the Distributed Optical Lever

In order to model the optomechanics of a cantilever-free probe, we have formulated a distributed optical lever model. Approximating a cantilever-free probe as rigid cone with base radius R resting on an infinite half plane of a compliant elastomer, Hertzian contact mechanics can be employed. Specifically, a cantilever-free probe is expected to behave as linear spring in which the spring constant k_{cf} is given by,

$$k_{cf} = 2RE, \quad (1)$$

with elastomer modulus E and taking the elastomer Poisson's ratio to be 0.5. It is important to note that the DLW written polymer has a modulus ~3 GPa[1], which is 10,000 times higher than the modulus of the PDMS film[2]. Because of this, internal deformation of the DLW-written structure is expected to be negligible. As depicted in Supplementary Figure 1a, the application of a force on the probe will indent it a distance δ_0 and cause a deformation δ of the surface at radial distance r from the center of the probe given by (From Eq. 3.42a in Ref. 3),

$$\delta(r) = \delta_0 \frac{2}{\pi}\left[\left(2-\frac{r^2}{R^2}\right)\sin^{-1}(R/r) + \frac{r}{R}\left(1-\frac{R^2}{r^2}\right)^{1/2}\right] \text{ for } \ r \geq R. \quad (2)$$

However, vertical motion of the surface will not lead to an appreciable change in the reflected light intensity. Instead, the slope of the surface will determine the angle of light reflection, so we define a local angle χ by,

$$\tan\chi = \frac{\partial\delta(r)}{\partial r}. \quad (3)$$

Taking the derivative of Eq. (2), we find

$$\tan\chi = \frac{2\delta_0}{R} f\left(\frac{r}{R}\right), \quad (4)$$

with

$$f\left(\frac{r}{R}\right) = \frac{2}{\pi}\left[-\frac{r}{R}\sin^{-1}\left(\frac{R}{r}\right) + \left(1-\frac{R^2}{r^2}\right)^{1/2}\right]. \quad (5)$$

Here, $f(r/R)$ is -1 at $r = R$ and asymptotes to 0 at $r \gg R$.

Having found a relationship between the motion of the probe and the slope of the interface, we sought to determine the optical consequences of probe motion. Assuming that the surface is being uniformly illuminated by the circular aperture that has an angular aperture β, we may compute the change in light intensity I relative to a maximum intensity I_{max} upon specular reflection from a flat surface with tilt angle χ (Supplementary Figure 1b). Specifically, this amounts to determining the overlapping region between two circles of radius $L\sin\frac{\beta}{2}$ whose center points are displaced by a distance $2L\sin\chi$ where L is the distance between the focal point and the objective (Supplementary Figure 1c). Specifically,

$$\frac{I(\chi)}{I_{max}} = \frac{2}{\pi}\cos^{-1}\left(\frac{\sin\chi}{2\sin\frac{\beta}{2}}\right) - \frac{\sin\chi}{2\,\pi\sin\frac{\beta}{2}}\sqrt{\left(4-\frac{\sin^2\chi}{\sin^2\frac{\beta}{2}}\right)}. \quad (6)$$

Taken together, the mechanical and optical models allow one to predict the optical contrast upon probe deformation. Specifically, Eq. (2) can be used to predict the deflection profile (Supplementary Figure 1d). Further, Eqs. 4, 5, and 6 may be combined to produce an expected I profile vs. r (Supplementary Figure 1e), plotted here with R = 3 μm and β = 22.5° (computed for an objective with a 0.28 numerical aperture in a medium with an 1.43 index of refraction). Using

this model, we can average the predicted intensity in a 15 μm diameter circle around a probe and compute the average I, which is found to be linear with δ_0 in the range $0 < \delta_0 < 1$ μm (Supplementary Figure 1f), an exciting result in terms of the translating this optical contrast to an imaging capability.

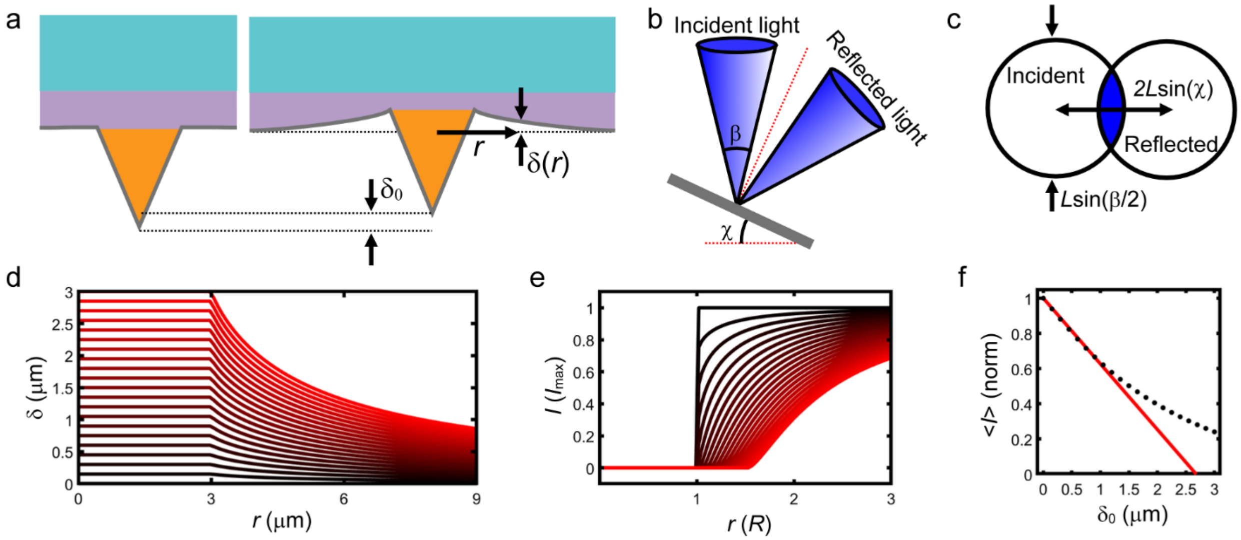

Supplementary Figure 1. Development and analysis of the distributed optical lever model. (a) Scheme showing a probe being indented a distance δ_0 which leads to the reflective surface at distance r being having a vertical translation $\delta(r)$. (b) Upon the surface being rotated by an angle χ, an incident light cone with angular aperture β is reflected. (c) The fraction of the reflected light that is captured is given by the intersection of two circles a distance L from the surface. (d) Calculated δ vs. r profiles with δ_0 varying from 0 (black) to 3 μm (red). Values are computed using Eq. 2 with the displacement under the probe being held constant. (e) Reflected light intensity I normalized by maximum intensity I_{max} calculated using Eqs. 4, 5, and 6. The color scale denotes δ_0 as in (d). The region beneath the probe is assumed to reflect no light due to the sharp nature of the conical probe. (f) Computed area-averaged intensity $\langle I \rangle$ in a 15 μm diameter circle around a probe deflected by δ_0. The curve is well approximated by a linear fit for $\delta_0 \leq 1$ μm with slope 0.37 μm^{-1}.

In addition, these expressions can be simplified in the limit of small deformations to produce an estimate for the expected deflection resolution of cantilever-free probes. Specifically, For small deformations (*i.e.* $\delta_0 \ll R$), the small angle approximation can be used to simplify Eq. (4) to find,

$$\chi \approx \frac{2\delta_0}{R} f\left(\frac{r}{R}\right). \tag{7}$$

Similarly, Eq. (6) can be linearized assuming χ is a small angle to find,

$$\frac{I(\chi)}{I_{max}} \approx \left(1 - \frac{|\chi|}{\pi \sin\frac{\beta}{2}}\right). \tag{8}$$

Combining Eqs. (7) and (8) leads to,

$$\frac{I\left(\frac{r}{R}, \delta_0\right)}{I_{max}} \approx \left(1 - \frac{2\delta_0}{\pi R \sin\frac{\beta}{2}}\left|f\left(\frac{r}{R}\right)\right|\right) \quad \text{for} \quad r \geq R. \tag{9}$$

Given the monotonic nature of $f(r)$, the point on the surface with the highest resolving power will be the region directly on the border of the conical probe (e.g. $r = R$). Thus, using this approximation, we may estimate the sensitivity of the distributed optical lever as,

$$\frac{\partial I}{\partial \delta_0}(r = R, \delta_0) = -\frac{2I_{max}}{\pi R \sin\frac{\beta}{2}} \tag{10}$$

Which suggests that for high performance, small probes, small numerical aperture objectives, and bright light sources will result in the highest sensitivity. Practically, the gain and exposure settings of the camera should be adjusting such that I_{max} corresponds to an intensity value of 1 in the camera. With this in mind, the bit depth N of the camera will determine the minimum resolvable intensity change ΔI_{cam} which correspondingly determines the resolution with an expected sensitivity of,

$$\Delta I_{cam} = \frac{I_{max}}{2^N}. \tag{11}$$

Combining Eqs. 9 and 10 allows one to estimate the minimum resolvable deflection $\Delta\delta_0$ as,

$$\Delta\delta_0 = \frac{\pi R \sin\frac{\beta}{2}}{2^{N+1}}, \tag{12}$$

which is ~1 nm for an 8-bit camera with R = 3 μm and β = 22.5°.

Supplementary Note 2. Estimation of Probe and Sample Deformation

As the conical probe itself is composed of a polymeric material, it is conceivable that tip deformation could limit resolution or otherwise interfere with the ability to quantitatively map sample topography. In analogy with cantilever-based AFM measurements, one can define the vertical z-piezo motion as ΔZ and the deflection of the backing layer (or in the case of cantilever-based AFM, the deflection of the cantilever) as δ_0. In addition to backing layer deformation, the probe tip can deform with magnitude δ_p in a manner that would reduce the total probe height h_p. Further, the sample itself can experience a deformation – or indentation – which can be defined as δ_s. These motions are interrelated by the expression,

$$\Delta Z = \delta_0 + \delta_s + \delta_p. \qquad (13)$$

In conventional cantilever-based AFM, $\delta_p \ll \delta_0$ as cantilevers are generally chosen to be much softer than the tip-sample spring constant. Further, for typical imaging applications, probe stiffness is chosen such that $\delta_s \ll \delta_0$, which simplifies calibration of the probe and facilitates quantitative topographical mapping. In contrast, nanomechanics measurements are often performed with $\delta_s \sim \delta_0$. Such sample deformation is a critical part of quantitatively evaluating the mechanics of the underlying sample.

In order to determine the importance of each of these deformations for cantilever-free AFM, we performed a series of finite element simulations. Specifically, we performed an axisymmetric simulation using COMSOL designed to match as closely as possible the experimental conditions. A 3 GPa conical probe with tip radius ρ = 100 nm, 6 μm height, and 3 μm bottom radius was positioned on a backing layer with 300 kPa modulus. A sample with modulus E_s was positioned in contact with the probe and then moved ΔZ towards the probe (Supplementary Figure 2a). This process allowed us to compute δ_s, δ_p, and δ_0 for a given ΔZ and E_s. Exploring $\Delta Z =$ 100 nm as a typical indentation, the deformation of the sample follows an expected distribution based upon E_s (Supplementary Figure 2b and Supplementary Table 1). Specifically, at $E_s >$ 1 GPa, nearly all of the deformation is localized in the backing layer while when $E_s <$ 10 MPa, the backing layer deforms very little and the sample is indented to a great extent. This leads us to conclude that, for this probe geometry, topographic imaging is possible when $E_s >$ 10 MPa as this region has a substantial backing layer deformation. Nanomechanical measurements require appreciable sample deformation and, thus, nanomechanical experiments should be possible when 1 GPa > $E_s >$ 10 MPa.

It is also important to consider how the contact area will change upon contact. These simulations provide an avenue to calculate contact area using the approximation that, in Hertzian contact between a spherical tip and a planar substrate, the contact radius a is given by,

$$a = \sqrt{\rho(\delta_p + \delta_s)}. \qquad (14)$$

This reflects that, while the maximum δ_p observed for this range is ~4% of ΔZ, the tip-sample contact area can also be increased through the deformation of the sample. Interestingly, the

smallest a is observed at high E_s and low ΔZ, showing that these conditions would be ideal for high resolution topographical imaging (Supplementary Figure 2c).

Supplementary Table 1. Finite element simulation results of mechanics of different sample modulus at z-piezo motion $\Delta Z = 100$ nm for a variety of sample moduli.

**Sample modulus** E_s	**Probe deformation** δ_p **(nm)**	**Backing layer deformation** δ_o **(nm)**	**Sample deformation** δ_s **(nm)**
300 kPa	0.01	0.67	99.33
1 MPa	0.02	2.19	97.79
3 MPa	0.06	6.28	93.65
10 MPa	0.18	18.5	81.36
30 MPa	0.44	41.04	58.52
100 MPa	1.00	68.66	30.35
300 MPa	1.57	83.97	14.46
1 GPa	2.48	91.66	5.86
10 GPa	3.89	95.42	0.69
100 GPa	4.17	95.76	0.07

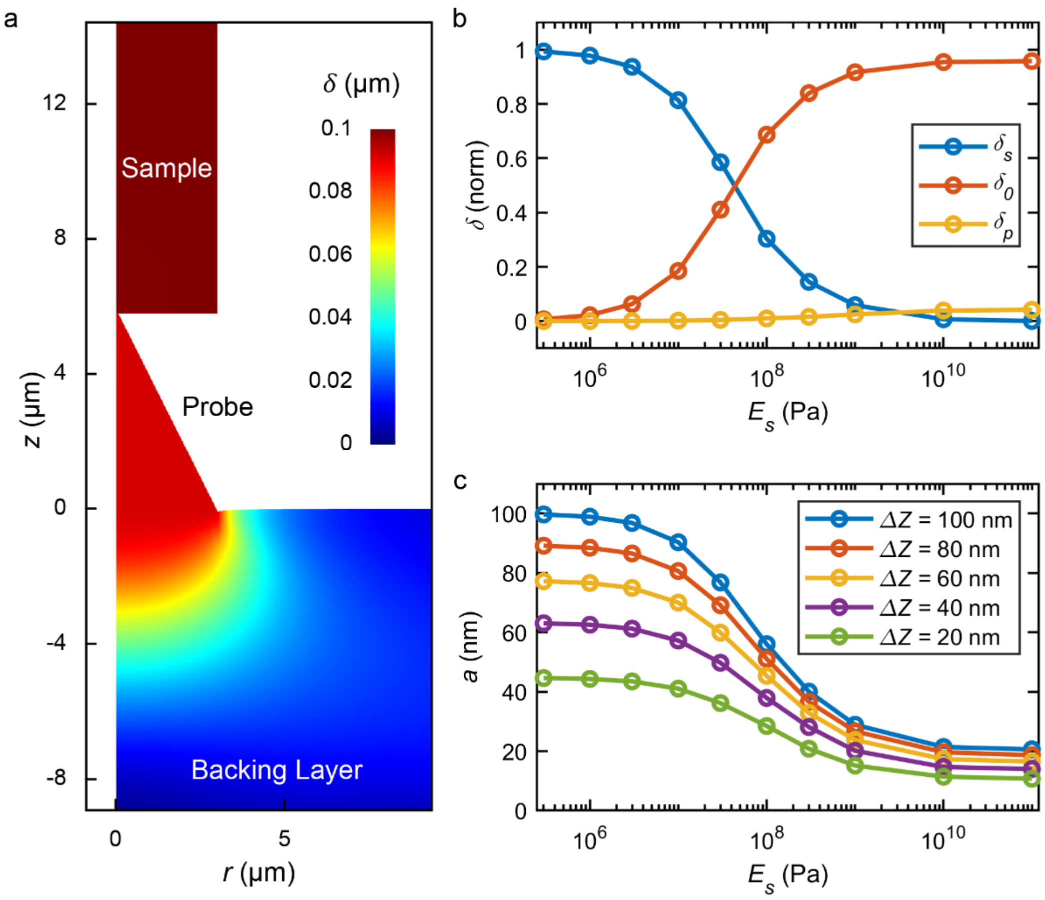

Supplementary Figure 2. Analysis of probe-sample mechanics. (a) Finite element simulation of the deformation δ associated with probe-sample contact of a cantilever-free probe and sample with sample modulus $E_s = 1$ GPa. (b) Simulated substrate indentation δ_s, backing layer deformation δ_o, and probe compression δ_p for a cantilever-free probe indenting a substrate with stiffness E_s. The simulation is performed by moving the sample $\Delta Z = 100$ nm. (c) Calculated probe-sample contact radius a as a function of E_s at different ΔZ.

Supplementary Note 3. Mechanical Characterization of Cantilever-free Probes

For the purpose of characterizing the mechanics of cantilever-free probes, we performed a series of experiments deforming cantilever-free probes using an AFM probe while observing them using an inverted microscope (Supplementary Figure 3a). Due to the probes themselves being much stiffer than the elastomeric substrate, the main role of the probe from a mechanical perspective is to determine the contact-area between the probe and the elastomeric support. Thus, as cylindrical and conical probes are expected to behave identically from a mechanical perspective, cylindrical probes were chosen for the sake of facile mechanical testing. Specifically, we prepared a cantilever-free array consisting of a series of DLW-printed cylinders, 6 μm tall with R ranging from 3 to 8 μm on a 14 μm thick PDMS film. The probe arrays were made reflective with a 30 nm aluminum coating.

In order to measure the mechanics of the cantilever-free probes, there were placed in combined AFM-inverted microscope system (MFP-3D Bio – Asylum Research). Mechanically, the probe arrays were characterized using an AFM probe (NSC16/No Al – MikroMasch). Initially, the probe was characterized by taking a thermal measurement of power spectral density (PSD) of the vibrational resonance of the probe and indenting a rigid surface (*i.e.* a glass slide) to determine the optical lever sensitivity. Together, these measurements allowed us to compute the cantilever spring constant k_c = 47.1 ± 0.3 N/m. Once calibrated, the AFM was used to image the region corresponding to a single cylinder as a means of positioning the AFM probe on the center of a cantilever-free probe. Subsequently, force-distance curves were performed and found to produce linear responses (Supplementary Figure 3b), which allowed us to compute the effective spring constant k_{eff} of the system as the slope of the force-indentation curve. As the cantilever-free probe and cantilevered AFM probe form two springs in series, the spring constant k_{cf} of the cantilever-free probe can be computed using,

$$k_{cf} = k_{eff} \cdot k_c \cdot \left(k_{eff} - k_c\right)^{-1}. \quad (15)$$

Interestingly, the k_{cf} was found to monotonically increase with R from 10.6 N/m to 31.1 N/m (Supplementary Figure 3c), in qualitative agreement with Eq. (1). This simple relationship underestimates k_{cf} as it does not take into account two important factors, (1) deformation of the comparatively rigid yet nanoscopic Al film and (2) the finite thickness of the elastomer film, which leads to confinement effects in a contact-area dependent manner[2]. By modeling this deformation using COMSOL (black line in Supplementary Figure 3c), which allows one to take these two factors into consideration, good agreement is found with experiment.

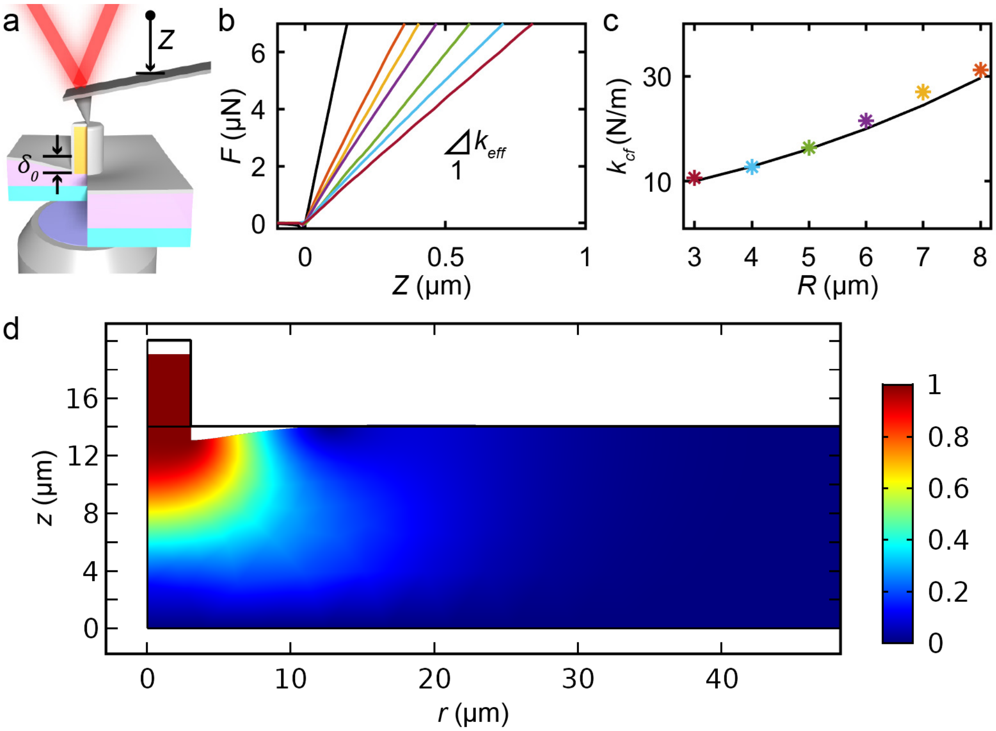

Supplementary Figure 3. Measured mechanics of cantilever-free probe arrays. (a) Schematic demonstrating the setup used to measure the mechanics and optics of cantilever-free probes. (b) Force *F* vs. displacement *Z* curves measured using an atomic force microscope (AFM) in contact with cylinders whose radii *R* are specified by the marker color. The black curve is the result of a *F* vs. *Z* curve on a rigid surface. (c) Corresponding spring constant k_{cf} of each cantilever-free probe with experimental values shown as points and finite element simulations shown as a line. The marker colors correspond to the colors in (b). (d) Finite element simulation of a 3 µm radius cylindrical punch experiencing a 10 µN force.

Supplementary Note 4. Estimation of Crosstalk

The deformation of one probe may lead to the motion of neighboring probes by virtue of the mechanical coupling through the common backing layer. This effect will lead to imaging artifacts as the sample height registered by a probe will be influenced by the height registered by neighboring probes. In order to explore this effect, we performed a three dimensional finite element simulation (COMSOL) in which a central probe was modeled as a cylinder such that a displacement boundary condition can be applied without any complications associated with internal deformation of the probe. Six conical probes were arranged in a hexagonal array around the central probe. The base radii of the conical probes and the radius of the central cylinder were all set to 3 μm and the probe-to-probe distance was set to 15 μm to match experimental values. Once set up, a 1 μm downward displacement was applied to the central cylinder and the resulting displacement of the system was computed (Supplementary Figure 4). Importantly, the neighboring probes were observed to move 0.29 μm, providing an estimate for crosstalk. It is also clear from the calculations that increasing probe-to-probe spacing will decrease crosstalk with near negligible crosstalk at ~30 μm. Thus, while crosstalk can be compensated for using post processing of images, it may also be removed at the cost of reduced sample throughput.

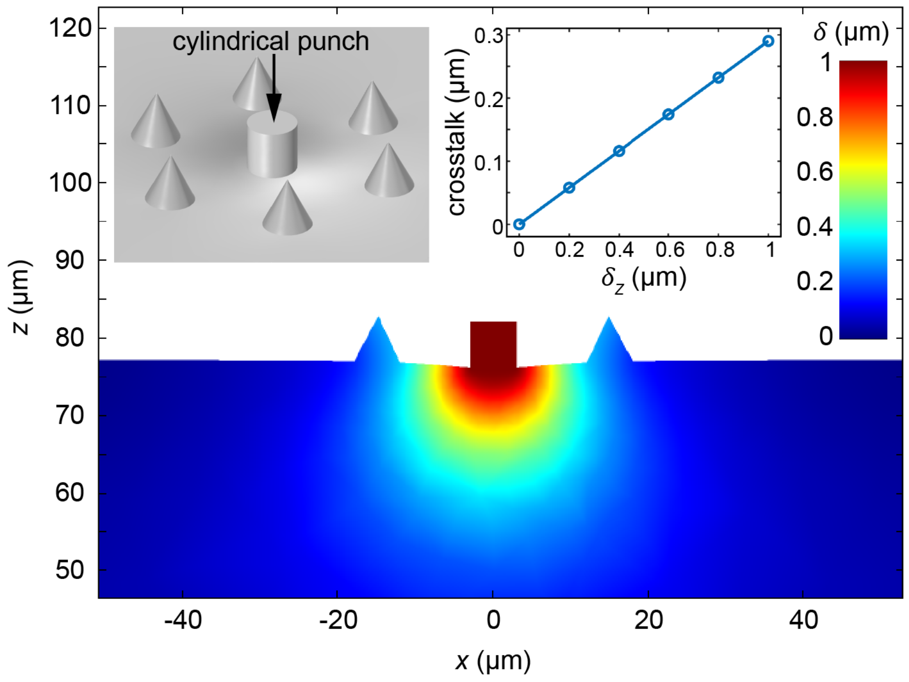

Supplementary Figure 4. Three dimensional finite element simulation of crosstalk by calculating δ of a seven probe array in which the central probe (modeled as a cylinder to reduce the impact of internal deformation) is deformed by 1 μm. Right inset shows the results of repeated simulations with different center probe deformations δ_z, showing the linearity of this relationship.

Supplementary Note 5. Optomechanics of Cantilever-free Probes

We sought to use the AFM platform to determine the degree to which deformation of cantilever-free probes was optically detectable. Thus, the AFM indentation experiments were repeated while observing the probe array through the sapphire wafer using a bright field optical microscope (Eclipse Ti – Nikon) with a high resolution camera (Point Grey Grasshopper GS3-US-32S4C-C) and 10× Olympus objective. As the force F on the probe increased, there was a drastic and visible change in the brightness in a region surrounding the probe (Supplementary Figure 5a). Repeating this measurement with multiple force set points revealed that this darkening increased in a monotonic fashion for $\delta_0 \leq 1.5$ μm, which comprised the whole tested range. To more quantitatively determine the relationship between the optical signal and the deformation, optical images were taken with fixed δ_0. An image taken while the AFM probe was out of contact was used as a baseline and subtracted from all images. Subsequently, each image was denoised using a low pass filter with a cutoff value at 65% of the maximum value of all the pixels over the region of interest (ROI) with a diameter of 15 μm centered around the probe and summed to produce an estimate of $\langle I \rangle$. In agreement with the distributed optical lever model, $\langle I \rangle$ was found to consistently increase across the whole range (Supplementary Figure 5b). The precision with which probe indentation δ can be measured was estimated to be 6 nm by multiplying the slope of the linear fit by the residual standard deviation in the range 0 to 100 nm, leading one to the conclusion that this simple approach can be used to achieve vertical resolutions better than 10 nm.

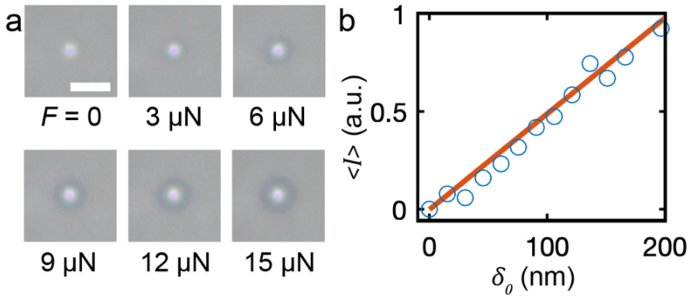

Supplementary Figure 5. Optomechanics of cantilever-free probe arrays. (a) Optical images of the visible deformation of the probe under increasing forces applied by AFM. Scale bar is 15 μm. Bright center regions in these images originate from the cylindrical nature of probes, in contrast with conical probes that are used for imaging. (b) Averaged intensity $\langle I \rangle$ of optical images from (a) vs. δ_0. The linear fit indicates that this optical metric can be used to determine δ_0. Prior to averaging, an image taken for δ_0 = 0 is subtracted from all frames.

Supplementary Note 6. Quantification of Repeatability

The ability of a measurement system to repeatability measure features is an important metric to consider. In an array-based system, this has two distinct facets. Specifically, one could describe the ability of a single probe to repeatability measure a feature (single probe repeatability) or one could describe the ability of multiple probes to measure the same feature (repeatability between probes). Measurements of known calibration samples provide a measure of these metrics. Specifically, the image taken of the fiducial arrow provides a useful dataset to explore these as we may identify a number of probes that image ostensibly identical regions of the arrow feature and compare their images (Supplementary Figure 6). In choosing four probes, (ID = 430, 562, 618 and 684), we find the step heights to be 119.8 ± 7.2 nm, 110.7 ± 6.6 nm, 113.1 ± 7.3 nm, and 115.0 ± 6.9 nm. These data can be analyzed to evaluate the single probe repeatability as the average coefficient of variation of these measurements, which was found to be ~6%, and the repeatability between probes as the coefficient of variation of the average found by each probe, which was found to be ~3%.

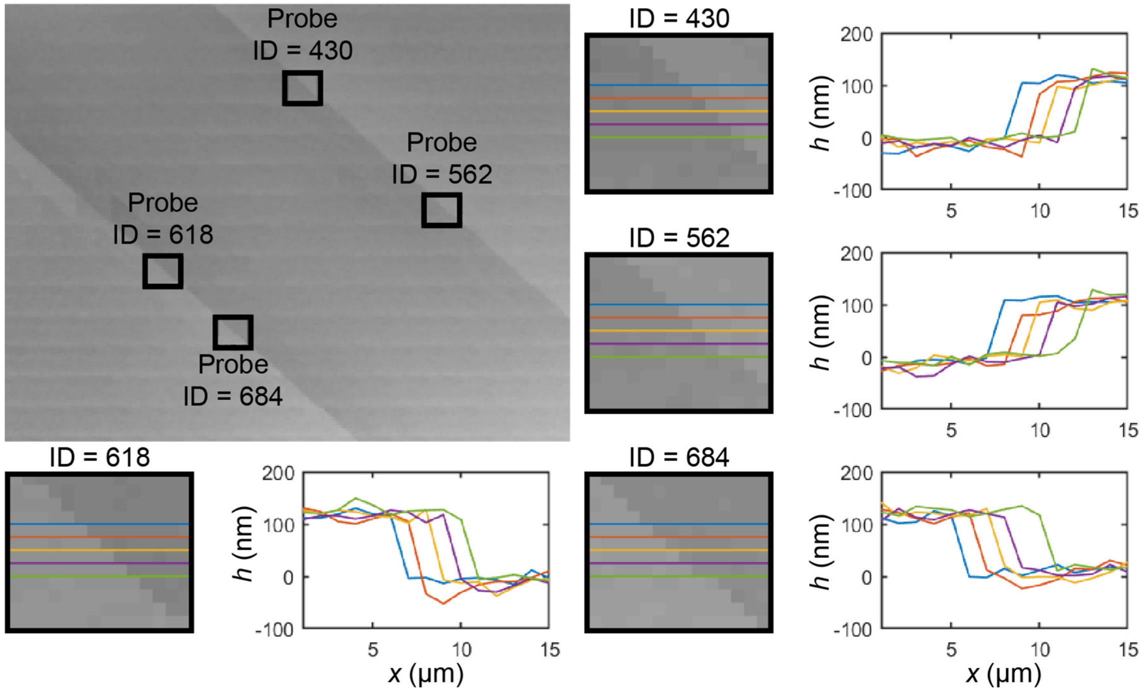

Supplementary Figure 6. Quantification of repeatability. Cantilever-free atomic force topographic image with zoomed in regions and line cuts corresponding to four probes (ID = 430, 562, 618 and 684).

Supplementary Note 7. Observing Lateral Forces with Cantilever-free Probes

We hypothesized that the cantilever-free architecture could be useful for other types of imaging beyond what is possible using conventional AFM. Specifically, conventional cantilever-based AFM is only sensitive to lateral forces in the direction perpendicular to the cantilever due to the limitations of four quadrant optical lever measurements. Further, due to the comparative stiffness of the torsional mode of typical AFM probes[4,5], the lateral force is typically neglected except when deliberately scanning laterally in contact mode to measure probe-sample friction using lateral force microscopy[6,7]. To study whether cantilever-free probes are sensitive to lateral forces, we indented a R = 3 μm cylinder at various displacements away from its center point. Analogous to a lateral force acting on the tip of a probe, an off-center normal force will produce a known torque about an axis running along the probe-backing layer interface (Supplementary Figure 7a). Critically, the optical signal detected changed markedly as the probe was moved along the pillar (Supplementary Figure 7b). Since a probe imaging a sample that is at an angle will produce lateral force, the ability to detect lateral forces in two dimensions provides an opportunity to directly measure the gradient of surface topography, an important new capability that could enable faster determination of sample topography.

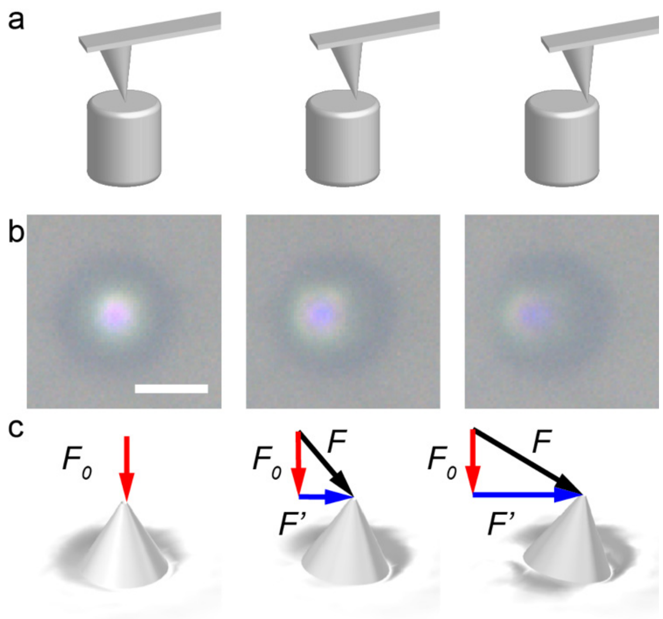

Supplementary Figure 7. Exploration of lateral forces. Lateral forces F' on the probes through (a) indenting cylindrical probes using an off-center force which leads to (b) visibly apparent anisotropic deformation. The torque applied to the probe is equivalent to (c) lateral forces acting at the tip of a conical probe. In all cases, the torque τ = 0, 8 and 16 μN·μm from left to right. Scale bar of the middle row is 10 μm.

Supplementary References

1. Alsharif, N. *et al.* Design and realization of 3D printed AFM probes. *Small* **14**, 1–6 (2018).
2. Li, L., Alsharif, N. & Brown, K. A. Confinement-induced stiffening of elastomer thin films. *J. Phys. Chem. B* **122**, 10767–10773 (2018).
3. Johnson, K. L. *Contact Mechanics*. (Cambridge University Press, 1987).
4. Turner, J. A. & Wiehn, J. S. Sensitivity of flexural and torsional vibration modes of atomic force microscope cantilevers to surface stiffness variations. *Nanotechnology* **12**, 322–330 (2001).
5. Huang, L. & Su, C. A torsional resonance mode AFM for in-plane tip surface interactions. *Ultramicroscopy* **100**, 277–285 (2004).
6. Cannara, R. J., Eglin, M. & Carpick, R. W. Lateral force calibration in atomic force microscopy: A new lateral force calibration method and general guidelines for optimization. *Rev. Sci. Instrum.* **77**, 053701 (2006).
7. Weymouth, A. J., Hofmann, T. & Giessibl, F. J. Quantifying molecular stiffness and interaction with lateral force microscopy. *Science* **343**, 1120–1122 (2014).
